# Evaluating quality of obstetric care in low-resource settings: Building on the literature to design tailor-made evaluation instruments - an illustration in Burkina Faso

**DOI:** 10.1186/1472-6963-10-20

**Published:** 2010-01-20

**Authors:** Florence Morestin, Abel Bicaba, Jean de Dieu Sermé, Pierre Fournier

**Affiliations:** 1 International Health Unit, CRCHUM (Research Centre of the University of Montreal Hospital Centre)/University of Montreal, 3875 Avenue Saint Urbain, Montreal, H2W 1V1, Quebec, Canada; 2 Burkinabé Public Health Association, 01 BP 3718, Ouagadougou 01, Burkina Faso; 3 Department of Social and Preventive Medicine, University of Montreal, 1430 Boulevard du Mont-Royal, Outremont, H2V 4P3, Quebec, Canada

## Abstract

**Background:**

There are many instruments available freely for evaluating obstetric care quality in low-resource settings. However, this profusion can be confusing; moreover, evaluation instruments need to be adapted to local issues. In this article, we present tools we developed to guide the choice of instruments and describe how we used them in Burkina Faso to facilitate the participative development of a locally adapted instrument.

**Methods:**

Based on a literature review, we developed two tools: a conceptual framework and an analysis grid of existing evaluation instruments. Subsequently, we facilitated several sessions with evaluation stakeholders in Burkina Faso. They used the tools to develop a locally adapted evaluation instrument that was subsequently tested in six healthcare facilities.

**Results:**

Three outputs emerged from this process:

1) A comprehensive conceptual framework for the quality of obstetric care, each component of which is a potential criterion for evaluation.

2) A grid analyzing 37 instruments for evaluating the quality of obstetric care in low-resource settings. We highlight their key characteristics and describe how the grid can be used to prepare a new evaluation.

3) An evaluation instrument adapted to Burkina Faso. We describe the experience of the Burkinabé stakeholders in developing this instrument using the conceptual framework and the analysis grid, while taking into account local realities.

**Conclusions:**

This experience demonstrates how drawing upon existing instruments can inspire and rationalize the process of developing a new, tailor-made instrument. Two tools that came out of this experience can be useful to other teams: a conceptual framework for the quality of obstetric care and an analysis grid of existing evaluation instruments. These provide an easily accessible synthesis of the literature and are useful in integrating it with the context-specific knowledge of local actors, resulting in evaluation instruments that have both scientific and local legitimacy.

## Background

Nearly all of the 500 000 maternal deaths worldwide every year occur in low- and middle-income countries (LMICs). Efforts to achieve the 5th Millennium Development Goal have been largely ineffective in regions with the highest maternal mortality, notably sub-Saharan Africa [1]. One strongly recommended strategy for reducing maternal deaths is to improve women's healthcare, especially during pregnancy and delivery [2]. Access to good obstetric care (OC) would prevent 50% to 70% of maternal deaths, reduce neonatal mortality by 10% to 15%, and substantially reduce the number of women living with sequelae of obstetric complications [3-5]. Good quality is essential not only in emergency OC, but also in basic OC, to detect complications early [5,6].

The first step in improving OC quality is evaluation, to identify problems. There are many freely available instruments for evaluating OC quality in LMICs. However, it is easy to lose one's way among these many instruments, whose evaluation approaches are quite diverse. Also, while healthcare may appear to be a well-defined field, there is nevertheless a certain amount of subjectivity in what is considered important in producing "quality" [7]. There is considerable variability in both the literature and the instruments, each of which studies OC quality from its own angle, focusing on specific elements: material resources, treatment protocols, women's satisfaction with services, etc. Each environment presents specific issues that may require evaluating some aspects of quality rather than others, such that ready-made instruments are not always appropriate. It is important to ensure that the instrument used in a given environment responds adequately to stakeholders' concerns, so that they will take ownership of the results [8,9]. Existing evaluation instruments provide a well-established scientific base upon which to build, having been tested already in their original environments. However, to this scientific base should be added "colloquial evidence" [10], i.e., the informal knowledge considered important by the stakeholders.

This article presents the process we followed to prepare a national evaluation of OC quality in Burkina Faso. We began with a review of existing evaluation instruments, which we then used to develop with stakeholders a locally appropriate evaluation instrument.

## Context

Burkina Faso is ranked next-to-last on the Human Development Index. Forty-six percent of its population lives under the poverty threshold, 75% of adults are illiterate, and life expectancy is only 51 years [11]. The maternal mortality ratio is 700 per 100 000 live births [2]. In rural areas, where 85% of the population lives, only 31% of deliveries occur in a healthcare facility [12], even though the geographic accessibility of OC has improved since the 1990s in the health districts--the healthcare system's first level and the designated point for managing OC. The government doubled the number of CSPSs (first-line health centres with at least one nurse, one auxiliary midwife, and one mobile health worker) and, beginning in 1994, to decentralize the management of obstetric and surgical emergencies, implemented in each health district a medical centre with a surgical unit (CMA) staffed by approximately 20 professionals.

In 2006 the Ministry of Health decided to subsidize OC and emergency neonatal services at 80% to encourage their utilization. Patients now pay only 20% of what they were previously required to pay, which had by far exceeded households' average health expenditures, to the point of being prohibitive for procedures such as caesareans [13-15]. This subsidy is an important step forward, but the Ministry of Health has cautioned that maternal mortality will be reduced only if parallel efforts are made to improve service availability and quality [16].

There appear to be numerous problems in this area. Several studies [17,18] have shown that providing more health facilities since the 1990s has not increased service utilization, most likely because of service quality problems. OC quality has not yet been systematically evaluated at the national level. However, a 2006 evaluation of health facilities' functionality in two districts [19] confirmed problems reported elsewhere [16,20]: shortages of qualified OC personnel in rural areas; lack of equipment, means of communication, and transport for evacuation referrals; very limited blood transfusion capacity, among others. As is often the case in LMICs, problems of quality are largely related to non-availability of resources; for this reason, quality of care is also measured in terms of availability. Because the population also perceives the quality to be poor, they may be dissuaded from using the healthcare system [21,22,18].

To document OC quality problems precisely and to support informed decision-making, the Burkinabé Public Health Association, working closely with the Ministry of Health, decided to evaluate OC availability and quality in the district health facilities (CMAs and CSPSs). They approached the University of Montreal/CRCHUM (Research Centre of the University of Montreal Hospital Centre) to help develop a context-adapted evaluation instrument.

## Methods

We carried out this study in five stages. First, we reviewed the literature to identify frameworks and evaluation instruments referring to OC quality in LMICs. This was not meant to be a systematic review; nevertheless, we conducted a broad search of both the scientific and the grey literature, as we expected most evaluation instruments to be found in the latter. We began in January 2007 by searching in Ovid MEDLINE for articles published since 1996. Using the combination of MeSH terms "*obstetrics" and "*quality of health care", we identified 32 articles. After reviewing their abstracts, we selected five of them; the remainder were excluded either because they were not about LMICs, dealt with other topics (e.g. postnatal care, analysis of delivery outcomes statistics in a specific area, etc.), were in languages other than English or French, or were not accessible. At that stage, we added some well-known seminal documents on OC in LMICs [23-25] as well as internal documents used by our team in ongoing projects in Africa [26]. Then we identified other relevant documents from the reference lists of this first set of documents. We continued with this snowball approach until reference lists of new documents only contained documents we had already identified. In total, we found 37 evaluation instruments, which are listed in Table 1. Nearly all of them were identified in the grey literature--twelve from EngenderHealth/AMDD [23,27], seven from Jhpiego [28,29], five from IMMPACT [24], four from the World Health Organization [25], four from Columbia University [30], and three from our own team [26]; only two were identified in scientific articles [31,32]. We submitted the list of instruments to an expert on OC in developing countries, who did not find any major instrument missing; still, given the profusion of instruments for evaluating OC quality in LMICs, there is no guarantee that our survey was totally comprehensive.

**Table 1:** Summary of characteristics of instruments for evaluating quality of obstetric care in low-resource settings

Instrument	Unit	Source(s) of information	Type of data	Structure (out of 19 items)	Process (out of 7 items)	Outcome (out of 6 items)
Room-by-room walk-through (Gill et al., 2005) [31]	F	O	QT	7	0	0

The walk-through with staff (EngenderHealth & AMDD, 2003a) [27]	F	O, St., R	QT	12	6	0

"Access to services and continuity of care" assessment form (EngenderHealth & AMDD, 2003b) [23]	F	St., O	QT	9	2	0

"Competent care" assessment form (EngenderHealth & AMDD, 2003b) [23]	F	St., O	QT	4	5	1

"Information & informed choice" assessment. form (EngenderHealth & AMDD, 2003b) [23]	F	St., O	QT	0	1	0

"Privacy, confidentiality, dignity, comfort & expression of opinion" assessment form (EngenderHealth & AMDD, 2003b) [23]	F	St., O	QT	3	2	0

"Facilitative supervision & management" assessment form (EngenderHealth & AMDD, 2003b) [23]	F	St., O	QT	9	0	0

"Information, training & development" assessment form (EngenderHealth & AMDD, 2003b) [23]	F	St., O	QT	4	0	0

"Supplies, equipment & infrastructure" assessment. form (EngenderHealth & AMDD, 2003b) [23]	F	O	QT	7	0	0

Registers & records review forms (EngenderHealth & AMDD, 2003b) [23]	F	R, MR	QT	1	0	0

Performance standards for EOC - Infection prevention (Jhpiego, USAID & Afghan MoH, 2005a) [28]	F	O	QT	3	0	0

Performance standards for EOC - Human, physical & material resources (Jhpiego, USAID & Afghan MoH, 2005b) [29]	F	O	QT	7	0	0

Performance standards for EOC - Support services (Jhpiego, USAID & Afghan MoH, 2005b) [29]	F	O	QT	7	0	0

Performance standards for EOC - Management systems (Jhpiego, USAID & Afghan MoH, 2005b) [29]	F	O, R	QT	5	0	0

Performance standards for EOC - Information, education & communication (Jhpiego, USAID & Afghan MoH, 2005b) [29]	F	O	QT	2	0	0

Skilled attendance index (Hussein et al., 2004) [32]	F	MR, R	QT	3	4	4

Health worker incentive survey (IMMPACT, 2007) [24]	F	St.	QT	6	0	0

Perceptions of quality of care - In-depth interview guide for users and non-users of maternity services (IMMPACT, 2007) [24]	F	P	QL	3	4	1

Perceptions of quality of care - Provider in-depth interview guide (IMMPACT, 2007) [24]	F	St.	QL	6	0	0

Perceptions of quality of care - Manager's interview guide (IMMPACT, 2007) [24]	F	St.	QL	9	0	0

Health facility staff interview guide (Maine et al., 1997) [40]	F	St.	QL	5	1	0

Supervision checklist for hospital/for health center (Maine et al., 1997) [40]	F	O	Co.	4	0	0

Performance standards for EOC - Care during normal labor, delivery & selected complications (Jhpiego, USAID & Afghan MoH, 2005b) [29]	C	O, MR, S	QT	3	7	0

Performance standards for EOC - Management of complications during pregnancy (Jhpiego, USAID & Afghan MoH, 2005b) [29]	C	O, MR, S	QT	1	7	0

Health facility questionnaire for case studies of women with obstetric complications (Maine et al., 1997) [40]	C	St., MR, P, Fm.	QT	2	4	2

Patient/Family Interview Guide, Case Studies of Women with OC (Maine et al., 1997) [40]	C	P or Fm.	QL	4	2	2

TRACE Maternal death assessment form (IMMPACT, 2007) [24]	C	MR or St.	QL	5	5	1

Facility-based maternal death review - Medical record extraction form (WHO, 2004) [25]	C	MR	QL	2	4	1

Facility-based maternal death review - Facility staff interview record (WHO, 2004) [25]	C	St.	QL	5	4	1

Case review form (EngenderHealth & AMDD, 2003b) [23]	C	MR	QL	1	2	4

EmOC client/family interview form (EngenderHealth & AMDD, 2003b) [23]	C	P or Fm.	Co.	1	3	1

Audits de décès maternels - Entrevue famille (Univ. of Montreal; CR-CHUM; Direction Régionale Santé Kayes-Mali 2006) [26]	C	Fm.	Co.	2	2	3

Audits de décès maternels - Entrevue centre de santé communautaire (Univ. of Montreal; CR-CHUM; DRS Kayes-Mali 2006) [26]	C	St.	Co.	3	4	3

Audits de décès maternels - Entrevue centre de santé de référence (Univ. of Montreal; CR-CHUM; DRS Kayes-Mali 2006) [26]	C	St.	Co.	4	4	3

Guidelines for conducting a near-miss case review (WHO, 2004) [25]	C	St., MR	Co.	9	6	1

Clinical audit - Case extraction form (WHO, 2004) [25]	C	MR	Co.	2	4	2

Client flow analysis - Client data form (EngenderHealth & AMDD, 2003b) [23]	C	O	Co.	0	2	0

Unit of observation: F = facility, C = case

Sources of information: O = observation, St. = staff, R = registers, MR = medical records, S = simulation, P = patient, Fm. = family

Type of data: QT = quantitative, QL = qualitative, Co. = combined

Second, from the literature collected, we inventoried the different components of OC quality and organized them into an exhaustive conceptual framework, as a tool to guide evaluation. "OC quality" is a broad and multifaceted concept. Evaluating it in practical terms requires very precise criteria focused on specific components; hence the value of a conceptual framework that details all the components of OC quality, each of which can serve as an evaluation criterion.

Third, we studied the 37 OC quality evaluation instruments using the descriptive-analytical method: we applied the same analytical framework to all the instruments to collect standardized information and to facilitate comparisons [33]. We presented the instruments in an analysis grid that followed the structure of our conceptual framework and highlighted the evaluation strategies and criteria used by each.

Fourth, in February and March 2007, we led a deliberative process with stakeholders in Burkina Faso to develop a locally relevant evaluation instrument. Deliberative processes are participative mechanisms for eliciting and combining "scientific" evidence from the literature with "colloquial" evidence from local stakeholders' experience to increase the probability of taking sound and acceptable decisions in a given context [10]. Specifically, a deliberative process involves bringing together stakeholders, presenting them with the scientific evidence on the subject of interest and engaging them in a discussion of how it can be integrated with their knowledge of the situation for informed and context-appropriate decision-making. Our working group was made up of six representatives of the Burkinabé Public Health Association and the Ministry of Health. In developing locally appropriate evaluation strategy and criteria, they used our conceptual framework for OC quality and considered a variety of situational factors. They operationalized the evaluation criteria in questions and response choices, using the analysis grid of existing instruments and their own expertise in OC in the CMAs and CSPSs.

Fifth, the evaluation instrument was finalized after being tested in two CMAs and four CSPSs, which allowed health professionals in the field to participate in the development process.

## Results

The results consist of three outputs: 1) a conceptual framework for OC quality; 2) an analysis grid of instruments for evaluating OC quality in LMICs; and 3) an evaluation instrument for OC quality in Burkina Faso.

### Conceptual framework for OC quality

**Figure 1:**
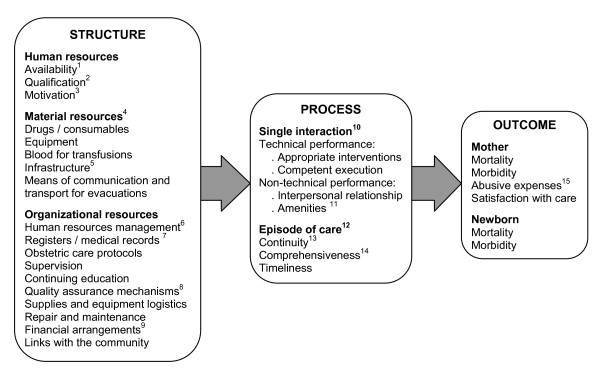
**Conceptual framework for the quality of obstetric care**. ^1^Number of human resources on staff and on duty 24 hrs/day, 7 days/week. ^2 ^*Qualification *is the fact, for example, of having a degree in medicine, midwifery, etc.; this is not to be confused with *competence*, which is expressed in the care process: qualification and competence are not automatically interrelated. ^3 ^A person's interest in pursuing the objectives of the organization for which he or she works. ^4 ^Should be available at all times, functional, and in sufficient quantity. ^5 ^Including buildings and support services (sterilization, laundry, etc.). ^6 ^E.g. team organization, job descriptions, regular payment of salaries, sanctions and rewards, etc. ^7^Should be in user-friendly formats and well maintained. ^8 ^E.g. review of cases having negative outcomes, collecting patient's opinions on services received, etc. ^9 ^Such that women are not required to pay anything *before *receiving obstetric services. ^10 ^Between the caregiver and the patient. ^11 ^Characteristics of the setting within which care is provided that help put the patient at ease (for example, not only are there curtains--a material resource--in the delivery room, but *the caregivers actually take care to close them *to protect the women's privacy). ^12 ^All of the single interactions, and how they are interconnected, from the beginning to the end of the patient's treatment. This looks at how services are organized. ^13 ^Within the health facility and, if the patient is referred, from one facility to another. ^14 ^All the services required are provided. ^15 ^Abusive fees charged by certain healthcare professionals, which are a flagrant sign of bad practices.

Even if the decision is taken to evaluate only *certain *aspects of OC quality that are considered the most important in a given context, this choice should be explained in relation to the entire set of possible evaluation criteria. This transparency is even more necessary when evaluation choices are negotiated between different stakeholders. In these circumstances, it is important to use a conceptual framework that is sufficiently exhaustive and operational to guide the selection of evaluation criteria. There is considerable variability in the evaluation instruments and in the literature on OC quality. In the absence of any consensus-supported conceptual framework on OC quality, we refined Donabedian's classical model [34,35], which considers three levels for evaluating the quality of care: *structure *(human, material, and organizational resources); *process *(the health services themselves); and *outcome *(the consequences of these services on patients). These levels follow a logical sequence: available resources, put into action, lead to activities that produce results. We inventoried the components of OC quality from the literature and organized them into these three categories, producing a comprehensive conceptual framework in which every item is a potential criterion for evaluating OC quality. Figure 1 presents this framework with brief explanations; detailed descriptions with references to the literature are in Additional File 1.

This conceptual framework does not judge the components' relative importance, which will, in any case, vary according to stakeholders' perspectives. Rather, it is a tool to support deliberation and the selection of the components to be evaluated in a given context.

The causal links between structure, process, and outcome are theoretical and not always verified in reality [34]. A good-quality structure has the potential to produce a good care process, but this potential may not be achieved. An evaluation focused only on outcomes, especially morbidity and mortality, does not discern to what extent these are due to quality of care rather than other factors, and therefore cannot guide decision-making for improving service quality. Thus, our review excluded instruments that measure *only *mortality or morbidity and nothing else, since they are not, strictly speaking, instruments for measuring OC quality. In short, if we use evaluation criteria from only one level, we cannot infer that the quality thus measured applies to the entire "chain of production" of OC.

### An analysis grid of instruments to evaluate OC quality in LMICs

Given the large number of OC quality evaluation instruments freely available in English, and often also in French, the problem is not a lack of material, but rather, navigating through it. Thus, we developed an analysis grid to record each instrument's content and evaluation strategy and applied it to the 37 instruments. Additional File 2 contains the full grid; Table 2 presents an extract. With respect to content, the grid reproduces the structure of our conceptual framework: each line is devoted to one component of OC quality, with x's marking the instruments that use it as an evaluation criterion. From the distribution of x's we can see on what level(s)--structure, process, or outcome--each instrument is focused. The grid also presents each instrument's broad evaluation strategies: unit of observation (i.e., facility or case management level); information sources (interviews with staff, patients, or families; reviews of medical records and registers; observation); and type of data gathered (quantitative, qualitative, or combined). Even though most instruments are designed to evaluate emergency OC, those whose unit of observation is a facility can also, because of their configuration, be used to evaluate basic OC.

**Table 2:** Extract from the analysis grid of instruments to evaluate the quality of obstetric care in low- and middle-income countries.

			Instrument:	**Access to services and continuity of care assmt. form (EngenderHealth & AMDD, 2003b) **[23]	**PQOC - Interview guide for users and non-users of maternity services (IMMPACT, 2007) **[24]	**Performance standards for EOC - Management of complications during pregnancy (Jhpiego, USAID & Afghan MoH, 2005b) **[29]	**Audits de décès maternels- Entrevue CSCOM (Univ. of Montreal, DRS Kayes-Mali 2006) **[26]
			**Unit of observation:**	**Facility**	**Facility**	**Case**	**Case**

			**Source(s) of information:**	**Staff, observation**	**Users & non-users**	**Observation, record, simulation**	**Staff**

			**Type of data:**	**Quantitative**	**Qualitative**	**Quantitative**	**Combined**

**STRUCTURE**	Human resources		Availability	**X**			**X**
		
			Qualification		**X**		**X**
		
			Motivation				
	
	Material resources		Drugs/consumables	**X**			
		
			Equipment	**X**			
		
			Blood for transfusions	**X**			
		
**'**		Infrastructure	Buildings	**X**	**X**		
			
			Support services				
		
			Means of communication & transport (evacuations)	**X**			
	
	Organizational resources		Human resources management				
		
			Registers & medical records	**X**		**X**	**X**
		
			Obstetric care protocols				
		
			Supervision				
		
			Continuing education				
		
			Quality assurance mechanisms				
		
			Supply logistics	**X**			
		
			Repair/maintenance				
		
			Financial arrangements	**X**	**X**		
		
			Links with community				

**PROCESS**	Single interaction	Technical perfor- mance	Appropriate interventions			**X**	**X**
			
			Competent execution			**X** (if observation)	
		
		Non-technical perfor- mance	Interpersonal relationship		**X**	**X**	
			
			Amenities		**X**	**X**	
	
	Episode of care		Continuity	**X**	**X**	**X**	**X**
		
			Comprehensiveness			**X**	**X**
		
			Timeliness	**X**	**X**	**X**	**X**

**OUTCOME**	Mother		Mortality				**X**
		
			Morbidity				
		
			Abusive expenses				
		
			Satisfaction with care		**X**		
	
	Newborn		Mortality				**X**
		
			Morbidity				**X**

The analysis grid can be used to prepare a new evaluation. For instance, if we want to assess quality at the level of OC process, we can easily locate in Table 2 the most comprehensive instrument for this purpose; if we want to evaluate the condition of buildings, a quick horizontal reading of the grid will show us two instruments for this. This allows us to go directly to the relevant instruments for detailed consultation and draw upon them as needed to produce a new instrument tailored to our specific context.

Table 1 summarizes the instruments, their evaluation strategies, and how many components of our conceptual framework they use as evaluation criteria; the specific components they use are noted in the full version of the analysis grid in Additional File 2. Table 1 reveals several trends. Instruments whose unit of observation is a facility essentially evaluate structural components; some also consider process components. All instruments whose unit of observation is a case focus on process. Of these, the majority also evaluate outcome, and half are very interested in structure (three components or more). Half of the instruments we surveyed use multiple sources of information. The choice of sources seems to be independent of the evaluation perspective and unit of observation. With respect to the type of data, instruments whose unit of observation is a case are more likely to collect unstructured responses that must then undergo qualitative analysis.

### An instrument to evaluate OC quality in Burkina Faso

The instrument developed to evaluate OC quality in Burkina Faso is presented in its entirety, in French, in Additional File 3. Table 3 summarizes its main features. The working group, made up of Burkinabé Public Health Association and Ministry of Health representatives, chose facilities as the unit of observation because evaluating managed cases required too many resources (time, qualified evaluators) to cover enough cases. Thus, the stakeholders are hoping to include a certain number of facilities in the evaluation. Likewise, they preferred to collect quantitative data because these are easier to process. The group's discussions on evaluation criteria were based entirely on the conceptual framework for OC quality. They felt it was essential to evaluate quality at the structure level to identify weaknesses at the source that could affect process and outcome. They selected most of the evaluation criteria for human and material resources. They considered the organizational resources criteria to be interesting but too sophisticated, given the more basic quality problems in Burkina Faso. Understanding that structural quality is necessary but insufficient, the working group wanted the evaluation to also touch upon certain aspects of process quality. Other dimensions, as well as the evaluation of outcomes, were set aside because they would require more resources than were available for this evaluation.

**Table 3:** Characteristics of the instrument for evaluating quality of obstetric care in Burkina Faso

				CMA	CSPS
			**Unit of observation:**	**Facility**	**Facility**

			**Sources of information:**	**Staff, dynamic observation, registers**	**Staff, observation, monthly reports**

			**Type of data:**	**Quantitative**	**Quantitative**

**STRUCTURE**	Human resources		Availability	**X**	**X**
		
			Qualification	**X**	**X**
		
			Motivation		
	
	Material resources		Drugs/consumables	**X**	
		
			Equipment	**X**	**X**
		
			Blood for transfusions	**X**	
		
		Infrastructure	Buildings	**X**	**X**
			
			Support services	**X**	
		
			Means of communication & transport for evacuations	**X**	**X**
	
	Organizational resources		Human resources management		
		
			Registers & medical records		
		
			Obstetric care protocols		
		
			Supervision		
		
			Continuing education		
		
			Quality assurance mechanisms		
		
			Supply logistics		
		
			Repair/maintenance		
		
			Financial arrangements	**X**	**X**
		
			Links with the community		

**PROCESS**	Single interaction	Technical performance	Appropriate interventions		
			
			Competent execution		
		
		Non-technical performance	Interpersonal relationship		
			
			Amenities		
	
	Episode of care		Continuity	**X**	**X**
		
			Comprehensiveness	**X**	
		
			Timeliness	**X**	**X**

**OUTCOME**	Mother		Mortality		
		
			Morbidity		
		
			Abusive expenses		
		
			Satisfaction with care		
	
	Newborn		Mortality		
		
			Morbidity		

The group used the analysis grid to identify instruments that dealt with each evaluation criterion retained. They consulted these instruments to see how they measured these criteria (questions, information sources) and to assess whether they were replicable in Burkina Faso, taking into consideration how OC is organized in this country and the uncertain availability and reliability of data. They recognized that the Burkina Faso instrument could not be as detailed as others because its purpose was to produce a first status report of OC quality in many facilities. The group was rarely able to reuse the questions exactly as they were in the existing instruments. Still, consulting them helped inspire and rationalize the process of developing the new instrument because the group was compelled to consider how to adapt the questions for Burkina Faso. It was also through this consultation that they adopted the "room-by-room walk-through" strategy [31], in which the facility evaluation visit follows the route taken by an obstetric case, thus allowing the evaluation to touch upon certain aspects of process even as it focuses on structure.

Finally, the questionnaire was tested in two CMAs and four CSPSs. Some questions were then rewritten to incorporate service providers' suggestions, to compensate for gaps in information sources, or to control for possible biases in staff responses. Field testing allowed us to identify the most reliable source of information for each question. Certain realities led us to adjust the "room-by-room walk-through" approach. In the CSPSs, where teams and infrastructure are limited, following the patients' route provided no additional information and interrupted the flow of the interview. So there we favoured "classic" staff interviews followed by visits to the maternity ward. In CMAs, the walk-through remains relevant; however, it is not possible to respect the patients' route strictly, because of social sensitivities: starting the evaluation visit with the ambulance drivers, even if they are the patients' first contact with the CMA, is not acceptable to the health workers.

## Discussion

### The need to tailor evaluation instruments to local contexts

Our experience highlights the fact that, even with the abundance of OC quality evaluation instruments specially designed for LMICs, it is rare that an existing instrument will work perfectly, as is, for a new evaluation project.

- Evaluation criteria: Our conceptual framework and our analysis grid highlight the multiplicity of possible criteria combinations. Chances are slim that an existing instrument's criteria set perfectly matches the issues under evaluation in a new context.

- Evaluation perspective and resource constraints: Many instruments were developed for case studies such as facility supervision or case management review and are therefore very detailed. In Burkina Faso, the evaluation must cover many facilities, but with a restrained budget that limits the time and human resources available for data collection and analysis. The evaluation questions therefore had to be simplified.

- Information sources: Documentary sources (registers, medical records) are less subject to desirability or memory biases than staff interviews. However, their availability and reliability vary from country to country, and an evaluation instrument that uses them may not be replicable elsewhere.

- Organizational and sociocultural realities: The logical reasoning underlying some evaluation instruments occasionally collides with local realities (e.g. the "walk-through").

Still, our experience also demonstrates that the literature (including evaluation instruments), if appropriately presented, can inspire and rationalize the development of a new instrument.

### Advantages of a synthesized presentation of the literature and evaluation instruments

The conceptual framework and the analysis grid of evaluation instruments proved useful as syntheses of the OC quality literature. The conceptual framework's components are not new; they come from the literature, where they are amply discussed. What is new, and what helps in rationalizing choices, as we saw with the Burkina Faso working group, is the framework's thoroughness and its structure based on Donabedian's three levels of evaluation of quality of care. Selecting criteria from a defined list involves justifying why the others are not retained.

Also, the visual representation of the relationships among the criteria and the levels (structure, process, or outcome) is a reminder that evaluation provides information on quality at the level evaluated, but not necessarily at the other levels. As for the analysis grid, our experience with the working group confirmed its ease of use for exploring the broad universe of existing instruments.

A key benefit of the conceptual framework and the analysis grid lies in their ability to present, in a synthesized, visual, and easily accessible way, the main elements from the scientific literature. This is especially important because the literature is still not readily available in many LMICs due to problems with Internet connections, cost of subscriptions to scientific journals [36], and language barriers for non-anglophones. Also, decision-makers and professionals (generally major stakeholders in the evaluations) are often unfamiliar with the literature and lack time to consult studies--hence the effectiveness of presenting them with syntheses [37,38] tailored to their requirements [39].

### The working group experience

We had two objectives in using a working group: to promote stakeholders' ownership of the evaluation instrument by involving them in its design, and to combine their informal knowledge of the evaluation context with scientific knowledge from the literature. The involvement of the Burkinabé Public Health Association, which launched the initiative, was a given. The Ministry of Health participated as the key decision-maker in matters of quality of care. Other stakeholders, notably funding agencies, also participate in these decisions, but to include them would have been cumbersome: an overly large working group is less effective [10], and developing an evaluation instrument is a detail-oriented project requiring members' active involvement. Service providers were not included in the working group for the same reasons, but some were consulted during field testing of the instrument.

The literature and the stakeholders' context-specific knowledge were easily integrated. Members of the working group appreciated the process and understood well and appropriated the lessons from the literature, such as the implications of evaluating OC quality at different levels, i.e., structure, process or outcome. The type of literature used--evaluation instruments and literature on OC--remained concrete and close to the health professionals' experience, and it was presented concisely and visually in a way that supported its direct, practical application, all of which facilitated its positive reception by the group.

There were no major disagreements around the development of the instrument, probably due to affinities between the institutions represented; some Association members have worked or are currently working for the Ministry. Minor disagreements were resolved pragmatically in the field testing, based on the feasibility of the evaluation options. The only difficulty was related to the availability of the group's members--busy professionals and decision-makers--for this process that involved several meetings and field visits outside the capital. We overcame this by dividing the group into working subgroups and providing continuous feedback on activities to the whole group.

## Conclusion

This experience of developing an instrument to evaluate OC quality and availability in Burkina Faso not only underscores the importance of tailoring instruments to the evaluation context, but also shows that existing instruments can inspire and rationalize the process. Two methodological tools produced during this experience could be useful to other evaluation teams: a conceptual framework for OC quality and an analysis grid of existing evaluation instruments. These tools synthesize the literature in a user-friendly format that supports integrating local stakeholders' informal knowledge with the literature to produce evaluation instruments that have both scientific and local legitimacy. In this case, using a deliberative process to integrate these two types of knowledge worked well. It will be important to follow the evaluation currently under way and how its results are used, to see how well this process fulfills its promise of promoting ownership by the local stakeholders.

## Abbreviations

OC: obstetric care; LMICs: low- and middle-income countries; CSPS: *Centre de Santé et de Promotion Sociale *(centre for health and social advancement); CMA: *Centre Médical avec Antenne chirurgicale *(medical centre with a surgical unit); CRCHUM: *Centre de recherche du Centre hospitalier de l'Université de Montréal *(Research Centre of the University of Montreal Hospital Centre).

## Competing interests

The authors declare that they have no competing interests.

## Authors' contributions

FM, PF, and AB designed the study. FM reviewed the literature on quality of obstetric care, developed the conceptual framework and analyzed the existing evaluation instruments. PF then reviewed the conceptual framework and analysis grid of the evaluation instruments. FM, with assistance from AB, facilitated the process of developing a new evaluation instrument in Burkina Faso, including the pre-test. JdDS played an important role in the development and pre-testing of the instrument. FM wrote the first draft of the manuscript which was then critically revised by PF, AB, and JdDS. All authors read and approved the final manuscript.

## Pre-publication history

The pre-publication history for this paper can be accessed here:

http://www.biomedcentral.com/1472-6963/10/20/prepub

## Supplementary Material

Additional file 1 **Literature review: Components of obstetric care quality**. Detailed description of the components of obstetric care quality inventoried from the literature. Click here for file

Additional file 2 **Analysis grid of instruments to evaluate the quality of obstetric care in low- and middle-income countries**. A grid recording the respective content and evaluation strategy of 37 instruments. Click here for file

Additional file 3 **Instrument to evaluate the availability and quality of obstetric care in Burkina Faso**. The instrument developed to evaluate OC quality in Burkina Faso presented in its entirety, in French. Click here for file
