# Supplementary material for: Evaluating quality of obstetric care in low-resource settings: Building on the literature to design tailor-made evaluation instruments - an illustration in Burkina Faso
Source: BMC Health Serv Res. 2010 Jan 20;10:20. doi: 10.1186/1472-6963-10-20 (PMC2837005; doi:10.1186/1472-6963-10-20)
Supplement: Additional file 1 — Literature review: Components of obstetric care quality. Detailed description of the components of obstetric care quality inventoried from the literature. [file 1472-6963-10-20-S1.DOC]

**Literature Review: Components of Obstetric Care Quality**

## 1. The quality of obstetric care, universally recognized as important…

Every year, 529 000 maternal deaths occur, nearly all of them in developing countries [1]. Access to obstetric care (OC) would prevent 50% to 70% of these deaths, reduce maternal mortality by 10% to 15%, and substantially reduce the number of women suffering for the rest of their lives from the consequences of obstetric complications—estimated at more than 10 million new cases per year [1-3]. For a long time, the focus has been on emergency OC; however, there appears to be a new consensus emerging on the importance of basic OC, since it is at this level that timely detection and management of complications would prevent them from becoming emergencies [1, 3-6]. Quality is therefore essential at all points along the OC continuum. Moreover, improving this quality must be a priority in low-risk maternity programs; before promoting the utilization of OC services on a large scale, their quality must be assured [7].

## 2. … but with no consensus on operational definition

An evaluation process requires an operational definition of quality from which specific evaluation criteria can be drawn. What are good quality OC services? Van Maanen (1984, cited by Sitzia and Wood, 1997 [8]) defines quality as the gap between what is and what should be. Yet “what should be” is a subjective judgment; hence the profusion of different concepts of quality of care [9]. This profusion is evident in the literature on OC quality. Most articles approach quality from a single angle, such as material resources, or treatment protocols, or women’s satisfaction with the care received. Breaking things down into various components in this way is essential for studying quality in practical terms and not just as a concept, but these one-dimensional approaches cannot capture the whole essence of quality. So, what is the combination of elements that makes up OC quality? Although some articles try to present a comprehensive picture of OC quality, there is considerable variety in what each considers to be its constituent components.

Given the lack of consensus on any conceptual framework for OC quality, we turned to a classic model developed for healthcare services as a whole, that of Donabedian [10, 11]. For Donabedian, there are three approaches for evaluating the quality of services, which are based on criteria related either to *structure* (human, material and organizational resources mobilized to provide services), *process* (the services themselves), or *outcome* (the consequences of these services on patients). These three levels are linked in a logical sequence: available resources, put into action, lead to activities that produce results. We inventoried all the elements that were presented in the literature as components of OC quality and organized them into these three categories, to produce as comprehensive a conceptual framework as possible.

Clearly, this inventory of the elements that make up OC quality makes no judgment on each one’s relative importance. In fact, such a judgment is impossible for two reasons, one practical and one epistemological. On one hand, much of the literature on OC quality comes from the “grey literature”[[1]](#footnote-2); these authors most often have an implicit concept of what quality is, and they rarely provide evidence to support why a certain element or set of elements would contribute more to quality than would others. On the other hand, if we accept quality as a construct that varies according to the perspective of the person conceiving it, such explanations would have no universal authority. This is why we do not consider our inventory of the constituent components of OC quality to be a final product that conveys precepts, but rather as a tool that, in the context of an evaluation and with the stakeholders involved, can be used to fuel discussion and support the negotiation of consensus on the respective importance of the elements that make up OC quality, for the purpose of selecting evaluation criteria.

## 3. Inventory of the constituent components of obstetric care quality

### a) Components related to structure

In a human resources intensive sector such as healthcare services, human resources have a major influence on quality [12]; however, this influence also depends on the environment in which they function, which is made up of material and organizational resources.

- **Human resources**

Clearly, their **availability** is a precondition. With respect to staffing, there should be sufficient human resources in place, both in terms of quantity and quality (according to the obstetric mission of the health facility being studied). This is a major issue, especially since the new international standard is that *all* deliveries should occur in settings that provide professional maternity care, and not only deliveries considered “high risk” according to earlier criteria, whose predictive value has now been called into question [1]. For day-to-day operations, human resources must be available 7 days a week, 24 hours per day [7].

Once available, the professionals responsible for OC quality must be productive. According to Koblinsky et al. (2006) [13], how well they perform will depend on their “competence” and motivation, and on available material resources (which we will discuss later).

Here, a clarification is in order. According to the Quebec Office of the French Language, while the terms “competence” and “qualification” tend to be used interchangeably, they designate two different concepts: “Professional competence is expressed in the application of knowledge, know-how, and behavioural skills in professional situations, whereas professional qualification relates to acquired skills and knowledge that are recognized according to standard employment categories” ([http://www.granddictionnaire.com](http://www.granddictionnaire.com/) [[2]](#footnote-3)). Competence is evaluated dynamically in action, and in our categorization, is related to process. On the other hand, as a characteristic of human resources, qualification relates to structure.

When a professional holds a **qualification**, we attribute to that person a certain type and level of knowledge and skill. In fact, a study in Senegal showed that midwives and physicians detect significantly more obstetric complications than do matrons and nurses [14]. However, the evaluation of qualification must be considered carefully in context because, for the same professional title, the standards for recruitment of candidates, the time spent in training and practice, and the content of the training will vary from one country to another and are often revised downward when there is a need to make up for low levels of professional coverage [13].

Finally, an individual’s professional **motivation** is defined as his (or her) commitment to pursue the objectives of the organization for which he works; this can be expressed in the person’s behaviour, but also in his thoughts and feelings about his work (cognitive and affective motivation) [12]. In the healthcare sector, where staff play the key role, quality depends upon their willingness to devote themselves to their responsibilities: all other components of quality are only necessary but insufficient conditions [1, 12].

- **Material resources**

These must be available in sufficient quantities, all the time, and be functional [15].

First, there are the resources required for providing care [7]:

- **drugs and consumables:** shortfalls in these have been associated with higher case fatality rates in hospitals providing essential OC services (The Prevention of Maternal Mortality Network, 1995, cited in Adeyi and Morrow, 1996 [4]);
- therapeutic and diagnostic **equipment**;
- **blood**, in referral facilities set up to do transfusions.

There is a variety of standardized lists of equipment, drugs and consumables required to provide OC services (see, for example, WHO 2006 [16]).

The quality of OC services also depends upon the **infrastructure** in which they are provided: condition and configuration of buildings, supply of water and electricity, support services (sterilization, laundry…) and furnishings of the maternity ward, etc. [7, 15].

Finally, OC quality can be measured in terms of the **means of communication and transport** for evacuation referrals to more specialized levels of care in cases of complications. While there are few scientific evaluations of their effectiveness, the experiences of various programs indicate a reduction in transfer times thanks to the use of communication methods at the primary care level and of motorized means of transport [3, 13].

- **Organizational resources**

These less tangible resources are sometimes overlooked. Yet they play a major role in operationalizing human and material resources [15].

**Human resources management** is a crucial factor in ensuring the steady availability of staff (organization of teams), defining their responsibilities (position descriptions), motivating and retaining them (salaries paid on time, systems of sanctions and rewards, etc.) [1, 5].

Other organizational resources contribute to quality by supporting professional competencies [17]:

- **registers and medical records**, in user-friendly formats; when carefully maintained, these support continuity in the care provided to the patient by different professionals and care teams, with information on observations made and treatments previously provided [4, 6].
- obstetric care **protocols**; in particular, protocols or tools that support decision-making regarding patient referrals, such as the partogram [3].
- **supervision**, which has effects (at least in the short term) on the performance of professionals and can be seen as a sign of support, increasing their motivation [13]. Conversely, as an example, a study in Senegal identified a lack of supervision as the main factor underlying professionals’ non-respect of treatment protocols [18].
- **continuing education**, to maintain and improve professional skills. Active learning methods have been proven more effective than theoretical teaching for achieving real change in professional practices [13]. However, it remains to be determined which methods will improve outcomes when “there is a dire lack of evidence on cost-effective approaches to improving competency, particularly in conditions where pre-service training is poor and working conditions inadequate.” ([1], pp. 133-134).
- **quality assurance mechanisms**; for example, systems for obtaining patients’ opinions, or reviews of cases with negative outcomes. Evidence on OC quality improvement due to techniques such as clinical audits is almost non-existent [13]. In a first-referral hospital in Senegal, after emergency OC protocols were implemented and followed up with clinical audits and staff supervision, the numbers of maternal morbidity diagnoses and of emergency interventions went up, and the case fatality rate went down; however, it is not possible to discern the extent to which these effects are due to the clinical audits, the supervision, or the protocols [18].

Other organizational resources are aimed at ensuring the availability and functionality of material resources. These include **logistics** related to supplies and equipment (stock management) and the **repair and maintenance** of equipment and buildings [17].

The quality of OC also requires **financial arrangements** that will guarantee that both OC and any transportation required for evacuations are available without delay [17]. Financial accessibility is considered either as a component of service quality or only as a factor that enables services to be received regardless of their quality [11]. Whatever the case, we prefer to stay out of this debate: we include here, specifically, financial arrangements made to ensure that women do not have to pay anything *before* receiving OC services or being evacuated.

Finally, among the organizational resources that make up OC quality, we can include “**functioning linkages between the health services and the community** to sustain interactions, communications and partnerships” ([17], p. 14).

### b) Components related to process

Donabedian’s conceptual framework for the healthcare process [10], while not specifically developed for OC, seems to be the most comprehensive. Two perspectives are considered: the single provider–patient interaction and the episode of care (that is, the entire set of single interactions and how they are linked, from the beginning to the end of the patient’s treatment).

- **Single interaction**

On this level, Donabedian distinguishes between technical and non-technical performance. Performance is a phenomenon situated very much downstream from qualification as defined above: it is the result of the interaction between caregivers’ skills and motivation, and the material and organizational resources available to them [13, 19].

**Technical performance** consists of applying science and medical technology in order to optimize benefits to the patient without increasing risks. Brook and Kosecoff (1988) [20] refined this concept by distinguishing two aspects:

- the appropriateness of the interventions. This is especially relevant to OC: specialized services must necessarily be provided to women presenting with complications; on the other hand, normal deliveries should not be over-medicalized [21]. Of particular concern is the systematic practice of interventions that evidence has shown to be of no use or best reserved to certain situations: enemas, perineal shaving, systematic episiotomies for first deliveries, administration of oxytocin to accelerate labour, etc. [6].
- the competent execution of interventions.

The evaluation of these two aspects is necessarily normative; it consists of assessing the interventions against international standards for OC management (see, for example, WHO 2006 [16]), which are themselves based on the medical knowledge in effect at a given moment in time.

For Donabedian, **non-technical performance** is observed in the management of the interpersonal relationship with the patient, to which amenities contribute, i.e., characteristics of the setting within which care is provided that help put the patient at ease (for example, not only are there curtains—a material resource—in the delivery room, but *the caregivers actually take care to close them* to protect the women’s privacy).

Long neglected in favour of a technical approach to quality (quite understandable given the urgency of preventing negative pregnancy outcomes), non-technical performance is beginning to be recognized as an important constituent component of OC quality [13, 15, 17, 22].

This importance emerges from the considerable emotional and cultural pressure associated with childbirth [21] and from the fact that patients see the interpersonal relationship with care providers as one of the major components of OC quality—a universal observation [8] confirmed for OC services by studies carried out, among others, in Benin [22] and in Ghana [23].

The quality of non-technical performance is assessed, in part, on the basis of relatively universal ethical standards of the health professions (patient information, confidentiality, etc.), but it also relates to patients’ expectations, which themselves are based on social standards. This raises the issue, rarely studied, of women’s preferences with respect to the delivery environment, procedures, characteristics of the care providers, etc. [13].

- **Episode of care**

Evaluating the quality of care within a broader time frame is particularly relevant when it comes to OC services, which unfold over periods of many hours, or even days, and involve a succession of single interactions. At the level of the episode of care, quality depends not only on the quality of single interactions, but also on the **continuity** of services (as much in one facility, as among the different facilities involved, in the case of a referral) and their **comprehensiveness** (all the services required are provided). A third dimension is crucial for OC quality: the **timeliness** of interventions once the patient is in the healthcare setting [7, 24, 25].

### c) Components related to outcome

The quality of OC services can be evaluated in terms of their consequences, which have to do with maternal and perinatal mortality and morbidity (since OC services have the unique characteristic of involving two individuals; [21]), abusive expenses charged to the women, and satisfaction with the care received.

- **Mortality**

Case studies are particularly well-suited to evaluating the link between a maternal or newborn death and the quality of OC services provided. When we look at consolidated outcomes measures, the link is more difficult to establish. We cannot use maternal mortality as the indicator because of the difficulty of attributing deaths to OC quality rather than to other factors. The recommended outcome indicator for assessing OC quality is the case fatality rate among women admitted to a facility with an obstetric complication. However, this indicator must be used with caution because it is also influenced by the woman’s condition on arrival at the facility. Moreover, its use is only relevant in assessing OC quality in *referral* hospitals [7, 26, 27].

Regarding newborns, it is acknowledged that respiratory distress (the cause of one-quarter of perinatal deaths) and birth trauma are generally due to poor management of labour and delivery [1, 28]. Here, again, without doing in-depth case studies, it is difficult to distinguish between poor management *before* and *after* the mother’s arrival at the facility.

- **Morbidity**

Morbidity has to do with short- or long-term complications related to poor quality OC services. A pre-existing problem, poorly managed, may leave sequelae (obstetric fistula from prolonged dystocic labour, sterility from postpartum infection, pain from a poorly repaired episiotomy or perineal tear, etc.). Or it may be that poor quality OC itself produces a problem (infection of perfusion or suture sites, hemorrhage caused by a caesarean, infection in a newborn due to a non-aseptic cord section, etc.) [1, 24].

However, as WHO points out, “[these pathologies] are difficult to quantify, owing to problems with definitions and inadequate [medical] records” ([1], p. 65). Moreover, for pre-existing complications that were not well managed, it is difficult to determine to what extent the sequelae were due to poor OC quality and to what extent they were unavoidable because of the woman’s condition before receiving care.

- **Abusive expenses charged to the women**

In countries that implemented the Bamako Initiative, one “outcome” of OC is the fee that women must pay for their treatment. As mentioned earlier, we do not wish to enter here into the debate on whether financial accessibility is a component of healthcare service quality. This being said, there is a specific type of expense that does relate to the evaluation of OC quality, because it is a flagrant sign of bad practice, and that is the charging of abusive unofficial fees by certain service providers [29].

- **Women’s satisfaction with care**

The three components we have just described can only be used to evaluate OC services when they are of *very poor* quality, because they set the bar very low: the fact that a woman and her child survived the delivery in good health without having been subjected to extortion does not indicate that the services were of good quality. Birth is a major event, both emotionally and culturally, that is laden with the expectations of women and their families, such that the *cultural* accessibility of OC services [30] assumes great importance. Therefore women’s opinions of what constitutes good quality OC services, which have long been overlooked, should be considered [13, 22]. This is even more important because the perception of service quality is a determining factor in the decision to deliver in a healthcare facility [23, 25]. Attempts to improve the quality of OC services that do not take into account women’s quality criteria may produce disappointing results in terms of utilization [6].

- - Our literature review completed, we are able to construct a conceptual framework for OC quality (figure). This conceptual framework can serve as a guide to evaluation, since each of its items is in itself a criterion for evaluating OC quality. Once selected according to their relevance in a given evaluation context, these criteria will need only to be operationalized in evaluation questions.

**Conceptual Framework for the Quality of Obstetric Care**

**PROCESS**

**Single interaction**

Technical performance:

. Appropriate interventions

. Competent execution

Non-technical performance:

. Interpersonal relationship

. Amenities

**Episode of care**

Continuity

Comprehensiveness

Timeliness

**OUTCOME**

**Mother**

Mortality

Morbidity

Abusive expenses

Satisfaction

**Newborn**

Mortality

Morbidity

**STRUCTURE**

**Human resources**

Availability

Qualification

Motivation

**Material resources**

Drugs / consumables

Equipment

Blood for transfusions

Infrastructure

Means of communication and transport for evacuations

**Organizational resources**

Human resources management

Registers / medical records

Obstetric care protocols

Supervision

Continuing education

Quality assurance mechanisms

Supplies and equipment logistics

Repair and maintenance

Financial arrangements

Links with the community

References

1. World Health Organization: **The world health report 2005 - make every mother and child count.** Geneva: WHO; 2005.

2. Bouvier-Colle M-H, Ouedraogo C, Dumont A, Vangeenderhuysen C, Salanave B, Decam C: **Maternal mortality in West Africa. Rates, causes and substandard care from a prospective survey.** *Acta obstetricia et gynecologica Scandinavica* 2001, **80:**113-119.

3. Murray S, Pearson S: **Maternity referral systems in developing countries: Current knowledge and future research needs.** *Social Science and Medicine* 2006, **62:**2205-2215.

4. Adeyi O, Morrow R: **Concepts and methods for assessing the quality of essential obstetric care.** *International Journal of Health Planning and Management* 1996, **11:**119-134.

5. EngenderHealth, AMDD-Mailman School of Public Health-Columbia University: **Quality improvement for emergency obstetric care. Leadership manual.** New York: EngenderHealth; 2003a.

6. Hulton LA, Matthews Z, Stones RW: **Applying a framework for assessing the quality of maternal health services in urban India.** *Social Science and Medicine* 2007, **64:**2083-2095.

7. Maine D, Akalin MZ, Ward VM, Kamara A: **The Design and Evaluation of Maternal Mortality Programs.** New York: Center for Population and Family Health, Columbia University; 1997.

8. Sitzia J, Wood N: **Patient satisfaction: a review of issues and concepts.** *Social Science and Medicine* 1997, **45:**1829-1843.

9. Haddad S, Roberge D, Pineault R: **Comprendre la qualité : en reconnaître la complexité.** *Ruptures, revue transdisciplinaire en santé* 1997, **4:**59-78.

10. Donabedian A: **The quality of care. How can it be assessed ?** *JAMA* 1988, **260:**1743-1748.

11. Donabedian A: **Defining and measuring the quality of health care.** In *Assessing quality health care - Perspectives for clinicians.* Edited by Wenzel R. Baltimore: Williams & Wilkins; 1992

12. Franco LM, Bennett S, Kanfer R, Stubblebine P: **Determinants and consequences of health worker motivation in hospitals in Jordan and Georgia.** *Social Science and Medicine* 2004, **58:**343-355.

13. Koblinsky M, Matthews Z, Hussein J, Mavalankar D, Mridha M, Anwar I, et al.: **Going to scale with professional skilled care.** *Lancet* 2006, **368:**1377-1386.

14. Dumont A, de Bernis L, Bouillin D, Gueye A, Dompnier J-P, Bouvier-Colle M-H: **Morbidité maternelle et qualification du personnel de santé : Comparaison de deux populations différentes au Sénégal.** *Journal de Gynécologie Obstétrique et Biologie de la Reproduction* 2002, **31:**70-79.

15. Gill Z, Bailey P, Waxman R, Smith JB: **A tool for assessing ‘readiness’ in emergency obstetric care: The room-by-room ‘walk-through’.** *International Journal of Gynecology and Obstetrics* 2005, **89:**191-199.

16. World Health Organization, United Nations Population Fund, UNICEF, The World Bank: **Pregnancy, childbirth, postpartum and newborn care: A guide for essential practice (updated second edition).** Geneva: World Health Organization; 2006.

17. World Health Organization: **Making pregnancy safer: The critical role of the skilled attendant. A joint statement by WHO, ICM and FIGO.** Geneva: World Health Organization; 2004.

18. Dumont A, Gaye A, Mahé P, Bouvier-Colle M-H: **Emergency obstetric care in developing countries: Impact of guidelines implementation in a community hospital in Senegal.** *BJOG* 2005, **112:**1264-1269.

19. Veloski J, Tai S, Evans AS, Nash DB: **Clinical vignette-based surveys: A tool for assessing physician practice variation.** *American Journal of Medical Quality* 2005, **20:**151-157.

20. Brook RH, Kosecoff JB: **Competition and quality.** *Health Affairs* 1988, **Summer 1988:**150-161.

21. Pittrof R, Campbell O, Filippi V: **What is quality in maternity care? An international perspective.** *Acta obstetricia et gynecologica Scandinavica* 2002, **81:**277-283.

22. Grossmann-Kendall F, Filippi V, De Koninck M, Kanhonou L: **Giving birth in maternity hospitals in Benin: testimonies of women.** *Reproductive Health Matters* 2001, **9:**90-98.

23. D'Ambruoso L, Abbey M, Hussein J: **Please understand when I cry out in pain: women's accounts of maternity services during labour and delivery in Ghana.** *BMC Public Health* 2005, **5:**11 pages.

24. EngenderHealth, AMDD-Mailman School of Public Health-Columbia University: **Quality improvement for emergency obstetric care. Toolbook.** New York: EngenderHealth; 2003b.

25. Thaddeus S, Maine D: **Too far to walk: maternal mortality in context.** *Social Science and Medicine* 1994, **38:**1091-1110.

26. Maine D, Bailey P: **Indicators for design, monitoring and evaluation of maternal mortality programs.** In *AMDD Project workshop*. Marrakech; 2001.

27. UNICEF: **Guidelines for monitoring the availability and use of obstetric services (second edition).** New York: UNICEF; 2003.

28. Murray S, Davies S, Kumwenda Phiri R., Ahmed Y: **Tools for monitoring the effectiveness of district maternity referral systems.** *Health Policy and Planning* 2001, **16:**353-361.

29. Y. Jaffré, Olivier de Sardan J.-P.: *Une médecine inhospitalière. Les difficiles relations entre soignants et soignés dans cinq capitales d'Afrique de l'Ouest.* Paris: Karthala; 2003.

30. Beninguisse G, Nikièma B, Fournier P, Haddad S: **L’accessibilité culturelle : une exigence de la qualité des services et soins obstétricaux en Afrique.** *African Population Studies* 2005, **19:**243-266.

1. Manuals for overseeing OC quality, guides for planners and managers of low-risk maternity programs, evaluation reports, international statements and strategic documents, guidelines, etc. [↑](#footnote-ref-2)
2. Search term = Qualification, index = Political and social economics; consulted April 2, 2007. [↑](#footnote-ref-3)
