# Supplementary material for: Evaluating quality of obstetric care in low-resource settings: Building on the literature to design tailor-made evaluation instruments - an illustration in Burkina Faso
Source: BMC Health Serv Res. 2010 Jan 20;10:20. doi: 10.1186/1472-6963-10-20 (PMC2837005; doi:10.1186/1472-6963-10-20)
Supplement: Additional file 3 — Instrument to evaluate the availability and quality of obstetric care in Burkina Faso. The instrument developed to evaluate OC quality in Burkina Faso presented in its entirety, in French. [file 1472-6963-10-20-S3.DOC]

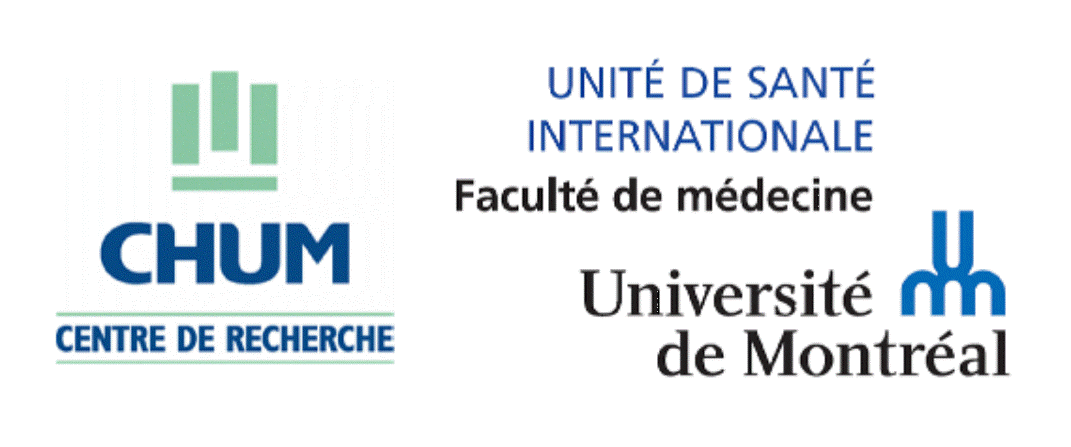

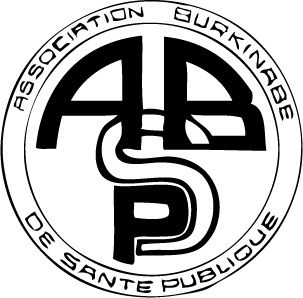

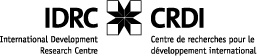

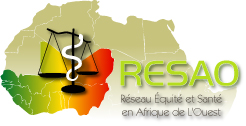


**Instrument d’évaluation de la disponibilité et qualité des soins obstétricaux (Burkina Faso)**

**Volet 1 : Disponibilité et qualité des soins obstétricaux d’urgence**

**dans les Centres Médicaux avec Antenne chirurgicale (CMA)**

Nom de l'enquêteur…………………………………………………………………….

Nom du superviseur……………………………………………………………………

Date de la visite………………………………

Mois couverts rétrospectivement *................................................…………………….

(* Indiquer quels sont les 3 mois calendaires précédant le mois durant lequel est réalisée la visite)

Région sanitaire……………………………………………………………………….

District sanitaire…………………….…………………………………………………

CMA de ……….………………………………………………………………………

**Cet instrument d’évaluation est administré successivement à :**

- **équipe de la maternité ;**
- **équipe du bloc opératoire ;**
- **techniciens de laboratoire ;**
- **caissier et gérant du dépôt/pharmacie ;**
- **équipe cadre de district (de préférence un responsable de la logistique);**
- **chauffeurs d’ambulance.**

Chaque équipe ou personne est rencontrée dans son propre service, ce qui permet de suivre plus ou moins les étapes de prise en charge d’une parturiente avec complication requérant une césarienne (l’ordre logique est cependant aménagé pour mieux se conformer à l’organisation du CMA).

**En début de visite : Demander les rapports mensuels du CMA pour les 3 derniers mois, et pré-remplir les informations sur le nombre d’évacuations (question 6) et le nombre de césariennes réalisées (question 13).**

**Maternité - Évaluation de la femme**

**Réunir plusieurs membres de l’équipe de la maternité pour faire l’entretien**

1. Quels sont les moyens de communication disponibles en cas d’urgences obstétricales ?

| Élément | Non disponible ou non fonctionnel | Disponible par intermittence * | Disponible en permanence |
| --- | --- | --- | --- |
|
| RAC |  |  |  |
| Téléphone fixe |  |  |  |
| Téléphone mobile appartenant au CMA |  |  |  |
| Téléphones mobiles appartenant aux membres du personnel |  |  |  |

* = périodes sans disponibilité durant les 3 mois passés, ou disponibilité seulement certains jours ou heures.

2. Interroger un membre du personnel qui fait les gardes sur place dans la maternité : Pendant les gardes, combien de temps en moyenne faut-il pour mobiliser un responsable de la maternité ?

|  | Toujours moins de 15 minutes | Parfois moins de 15 minutes | Toujours plus de 15 minutes | Parfois pas du tout mobilisable |
| --- | --- | --- | --- | --- |
| Gardes de nuit en semaine |  |  |  |  |
| Gardes du weekend |  |  |  |  |

3. Les ressources matérielles essentielles pour poser le diagnostic de la femme sont-elles disponibles dans la maternité et fonctionnelles ? (Interroger et observer)

| Élément | Non disponible | Disponible mais non fonctionnel | Disponible et fonctionnel | |
| --- | --- | --- | --- | --- |
|  |  |  | Oui | Nombre |
| Table d’examen |  |  |  |  |
| Stéthoscope obstétrical |  |  |  |  |
| Tensiomètre |  |  |  |  |
| Thermomètre |  |  |  |  |
| Mètre ruban |  |  |  |  |
| Spéculum |  |  |  |  |

4. Si l’évaluation de la femme indique qu’il faut pratiquer une césarienne - Pendant les gardes, combien de temps en moyenne faut-il pour mobiliser l’équipe du bloc (chirurgie et anesthésie) ?

|  | Toujours moins de 30 minutes | Parfois moins de 30 minutes | Toujours plus de 30 minutes | Parfois pas du tout mobilisable |
| --- | --- | --- | --- | --- |
| Gardes de nuit en semaine |  |  |  |  |
| Gardes du weekend |  |  |  |  |

5. Si l’évaluation de la femme indique qu’une transfusion sanguine est requise - Pendant les gardes, combien de temps en moyenne faut-il pour mobiliser un technicien de laboratoire ?

|  | Toujours moins de 30 minutes | Parfois moins de 30 minutes | Toujours plus de 30 minutes | Parfois pas du tout mobilisable |
| --- | --- | --- | --- | --- |
| Gardes de nuit en semaine |  |  |  |  |
| Gardes du weekend |  |  |  |  |

6. Nombre d’évacuations liées à la grossesse et l’accouchement durant les 3 derniers mois (information à relever dans les rapports mensuels du CMA) :

- Faire préciser quelles sont les évacuations comptabilisées dans les rapports mensuels :

| Évacuations depuis le CMA, vers un CHR* ou CHN** |  |
| --- | --- |
| Évacuations depuis les CSPS***, vers le CMA |  |
| Si pratiqué : évacuations depuis les villages, vers le CMA |  |

* Centre hospitalier régional ; ** Centre hospitalier national; *** Centre de santé et de promotion sociale.

- Relever le nombre de femmes évacuées :

| Mois | Nombre |
| --- | --- |
| Mois 1 |  |
| Mois 2 |  |
| Mois 3 |  |
| **Total** |  |

**Bloc opératoire**

**Réunir plusieurs membres de l’équipe du bloc pour faire l’entretien**

| 7. Personnel en poste | Nombre |  | Expérience totale : pratique depuis quelle année ? | Ancienneté dans le CMA : en poste depuis quelle année ? |
| --- | --- | --- | --- | --- |
| Médecins formés en chirurgie d'urgence |  | 1 | * |  |
|  |  | 2 | * |  |
| Aides anesthésistes |  | 1 |  |  |
|  |  | 2 |  |  |
| Aides chirurgiens |  | 1 |  |  |
|  |  | 2 |  |  |
|  |  | 3 |  |  |

* indiquer seulement les années d’expérience depuis la formation à la chirurgie

8. Est-ce qu’on a dû renoncer à faire au moins une césarienne au cours des 3 derniers mois, par manque de personnel clef ?  OUI  NON

9. Si oui, combien de fois : ………..

10. Si oui, absence de quel(s) personnel(s) :

| Médecin formé en chirurgie d'urgence |  |
| --- | --- |
| Aide chirurgien |  |
| Aide anesthésiste |  |

11. Est-ce qu’on a dû renoncer à faire fonctionner le bloc au cours des 3 derniers mois, pour des causes matérielles ?  OUI  NON

12. Préciser :

| Élément dont l’insuffisance a empêché le fonctionnement du bloc durant les 3 derniers mois | Manquant | Non fonctionnel / périmé | Nombre de jours de pannes / de rupture de stocks * |
| --- | --- | --- | --- |
|  |  |  |  |
|  |  |  |  |
|  |  |  |  |
|  |  |  |  |

* Interroger l’équipe et si besoin, confirmer les dates en consultant les registres du bloc.

13. Nombre de césariennes réalisées durant les 3 derniers mois :

- Voir d’abord les rapports mensuels du CMA et remplir la colonne « total » à droite du tableau.

- Puis voir les registres du bloc (registres des anesthésistes ou registre des protocoles opératoires), et relever le nombre de césariennes réalisées chaque mois, selon le moment du jour et la présence du médecin à compétence chirurgicale.

|  | Pendant la journée | | Pendant les gardes de nuit (17h-7h) | | **Total** |
| --- | --- | --- | --- | --- | --- |
|  | Avec médecin | Sans médecin | Avec médecin | Sans médecin |  |
| Mois 1 |  |  |  |  |  |
| Mois 2 |  |  |  |  |  |
| Mois 3 |  |  |  |  |  |
| **Total** |  |  |  |  |  |

- Vérifier les totaux sur place, pour repérer d’éventuelles erreurs de remplissage.

**Salle de post-op**

**Poursuivre la visite avec un professionnel de l’équipe du bloc**

14. Ressources matérielles essentielles à l’accueil des femmes césarisées en post-op : sont-elles disponibles et en bon état ? (Observer et noter les nombres dans chaque catégorie)

| Élément | Nombre  total | Nombre selon l’état * | | |
| --- | --- | --- | --- | --- |
|  |  | Bon | Moyen | Mauvais |
| Lits avec matelas |  |  |  |  |
| Toilettes à proximité de la salle de suite de couches |  |  |  |  |
| Sources d’eau potable dans l’enceinte du CMA |  |  |  |  |

* État des lits :

- matelas recouvert de skaï = bon

- skaï troué par endroits = moyen

- pas de skaï = mauvais

État des toilettes : jugé selon la propreté

Source d’eau :

- Si non fonctionnelle, noter zéro

- Robinet ou pompe = bon

- Puits grand diamètre aménagé = moyen

- Puits grand diamètre non aménagé = mauvais

**Laboratoire**

**Réunir plusieurs membres de l’équipe du laboratoire pour faire l’entretien**

15. Nombre de techniciens de laboratoire : …………..

16. Durant les 3 derniers mois, y a-t-il eu des jours sans aucun technicien présent au laboratoire ?

OUI  NON

17. Durant les 3 derniers mois, est-ce que certaines demandes de sang n’ont pas pu être satisfaites :

OUI  NON

18. Si oui, proportion de demandes non satisfaites :

| Moins d’une sur 10 |  |
| --- | --- |
| Entre une sur 10 et une sur 4 |  |
| Plus d’une sur 4 |  |
| Aucune transfusion réalisée au CMA |  |

19. Si oui, cocher la ou les raisons qui étaient en cause :

| Aucun service de transfusion dans le CMA |  |
| --- | --- |
| Manque de sang du groupe demandé |  |
| Manque de matériel / réactifs |  |

20. Pour les transfusions sanguines faites au CMA, d’où provient le sang le plus souvent ?

| Stock de sang dans le CMA |  |
| --- | --- |
| Transfusion directe |  |

**Tarification**

**Interroger le caissier et le gérant du dépôt/pharmacie**

21. Pratiquez-vous un système de paiement forfaitaire pour la césarienne :  OUI  NON

22. Si oui, montant du forfait : …….……… FCFA

23. Si paiement non forfaitaire, quels sont les tarifs ?

| Acte de césarienne |  |
| --- | --- |
| Kit opératoire |  |
| Montant moyen des ordonnances hors kit |  |

24. Moment du paiement par la femme ou sa famille :

- Si tarification par forfait :  Paiement avant césarienne  Paiement après césarienne

- Si tarification hors forfait, cocher les réponses appropriées :

| Moment du paiement : | Avant césarienne | Après césarienne |
| --- | --- | --- |
| Paiement de l’acte |  |  |
| Paiement du kit opératoire |  |  |

**Système de référence-évacuation**

**Moyens de communication : Interroger un membre de l’équipe cadre de district (de préférence un responsable de la logistique)**

25. Possibilités de communication avec les CSPS du district :

| Nombre total de CSPS dans le district |  |
| --- | --- |
| Nombre de CSPS joignables en permanence |  |
| Nombre de CSPS joignables de manière intermittente |  |

Uniquement si la personne interrogée hésite sur le nombre : passer en revue CSPS par CSPS.

| Nom du CSPS | Joignable en permanence | Joignable de manière intermittente |
| --- | --- | --- |
|  |  |  |
|  |  |  |
|  |  |  |
|  |  |  |
|  |  |  |
|  |  |  |
|  |  |  |
|  |  |  |
|  |  |  |
|  |  |  |
|  |  |  |
|  |  |  |
|  |  |  |
|  |  |  |
|  |  |  |
|  |  |  |
|  |  |  |
|  |  |  |
|  |  |  |
|  |  |  |
|  |  |  |
|  |  |  |
|  |  |  |
|  |  |  |
|  |  |  |
|  |  |  |
|  |  |  |
|  |  |  |
|  |  |  |
|  |  |  |

**Moyens de transport : interroger le ou les chauffeurs d’ambulance**

26. Le CMA dispose-t-il d’au moins une ambulance :  OUI  NON

27. En cas d’urgence, une ambulance du CMA est-elle rapidement mobilisable 24h/24 :

OUI  NON

28. Durant les 3 derniers mois, est-il arrivé qu’aucune ambulance du CMA ne puisse être utilisée, à cause de pannes ?  OUI  NON

29. Si oui : pendant combien de jours …………….

30. Si l’ambulance du CMA est indisponible, peut-on facilement mobiliser :

|  | OUI | NON | N’a jamais été nécessaire |
| --- | --- | --- | --- |
| Une ambulance du CHR ou CHN |  |  |  |
| Une ambulance de CSPS |  |  |  |

31. Quels sont les trajets d’évacuation faits par l’ambulance du CMA :

| Villages – CMA |  |
| --- | --- |
| CSPS – CMA |  |
| CMA – CHR (préciser lequel : ………………………) |  |
| CMA – CHN (préciser lequel : ………………………) |  |
| CSPS – CHR (préciser lequel : ………………………) |  |
| CSPS – CHN (préciser lequel : ………………………) |  |

32. Combien de CSPS sont inaccessibles pour l’ambulance durant la saison sèche, durant l’hivernage ?

|  | Saison sèche | Hivernage |
| --- | --- | --- |
| Nombre de CSPS accessibles |  |  |
| Nombre de CSPS inaccessibles |  |  |

Uniquement si le chauffeur hésite sur le nombre : passer en revue CSPS par CSPS

| Nom du CSPS | Accessible durant saison sèche | Accessible durant hivernage |
| --- | --- | --- |
|  |  |  |
|  |  |  |
|  |  |  |
|  |  |  |
|  |  |  |
|  |  |  |
|  |  |  |
|  |  |  |
|  |  |  |
|  |  |  |
|  |  |  |
| Nom du CSPS  (suite) | Accessible durant saison sèche | Accessible durant hivernage |
|  |  |  |
|  |  |  |
|  |  |  |
|  |  |  |
|  |  |  |
|  |  |  |
|  |  |  |
|  |  |  |
|  |  |  |
|  |  |  |
|  |  |  |
|  |  |  |
|  |  |  |
|  |  |  |
|  |  |  |
|  |  |  |
|  |  |  |
|  |  |  |
|  |  |  |

33. Pour les évacuations avec une ambulance du CMA : la femme ou sa famille doivent-elles effectuer un paiement avant que l’évacuation soit faite ?

|  | Oui | Non |
| --- | --- | --- |
| Évacuation depuis villages ou CSPS, vers le CMA |  |  |
| Évacuation depuis le CMA, vers CHR ou CHN |  |  |


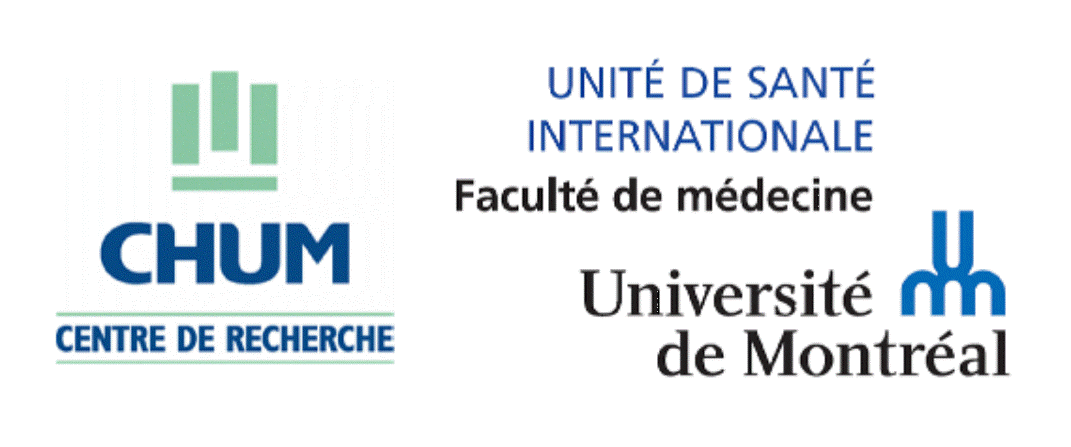

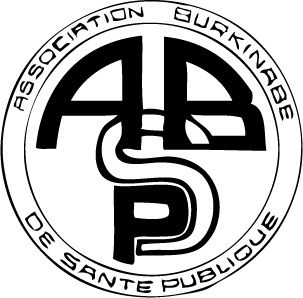

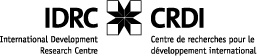

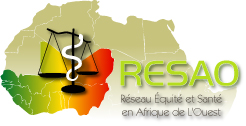


**Instrument d’évaluation de la disponibilité et qualité des soins obstétricaux (Burkina Faso)**

**Volet 2 : Disponibilité et qualité des soins obstétricaux**

**dans les Centres de Santé et de Promotion Sociale (CSPS)**

Nom de l'enquêteur…………………………………………………………………….

Nom du superviseur……………………………………………………………………

Date de la visite………………………………

Mois couverts rétrospectivement *................................................…………………….

(* Indiquer quels sont les 3 mois calendaires précédant le mois durant lequel est réalisée la visite)

Région sanitaire……………………………………………………………………….

District sanitaire…………………….…………………………………………………

CSPS de ……….………………………………………………………………………

**Administrer cet instrument à l’infirmier chef de poste et/ou au responsable de la maternité.**

**Accessibilité géographique**

1. Distance moyenne entre les villages et le CSPS : …….. km

**Ressources humaines**

| 2. Personnel en poste | Nombre | Expérience totale : pratique depuis quelle année ? | Ancienneté dans le CSPS : en poste depuis quelle année ? |
| --- | --- | --- | --- |
| IDE* (femme) |  |  |  |
| IDE (homme) |  |  |  |
| IB** (femme) |  |  |  |
| IB (homme) |  |  |  |
| Sage-femme |  |  |  |
| Maïeuticien |  |  |  |
| Accoucheuse auxiliaire |  |  |  |
| Matrone |  |  |  |
| AIS*** (femme) |  |  |  |
| AIS (homme) |  |  |  |

* IDE = infirmier diplômé d’État; ** IB = infirmier breveté; *** AIS = agent itinérant de santé.

3. Quel membre du personnel est responsable de la maternité ? (Indiquer son titre professionnel : IDE, IB, SFE, etc.) ……………………………………

4. A été absent durant les 3 derniers mois (pendant plus de 24 heures)?  OUI  NON

5. Si oui, nombre de jours d’absence : ……….

6. Quel membre du personnel supervise les soins obstétricaux en cas de complication ? (Indiquer son titre professionnel : IDE, IB, SFE, etc.) ……………………………………

7. A été absent durant les 3 derniers mois (pendant plus de 24 heures) ?  OUI  NON

8. Si oui, nombre de jours d’absence : ………...

9. Quels autres membres du personnel pratiquent des accouchements ? (Indiquer leurs titres professionnels : IDE, IB, SFE, etc.) ……………………………………………………

10. Est-ce qu’au moins un agent qualifié pour pratiquer les accouchements est à proximité du CSPS :

- la nuit (en semaine):  OUI  NON

- le week-end :  OUI  NON

**Ressources matérielles pour le suivi du travail et l’accouchement**

| 11. Éléments  (interroger et observer) | Non disponible | Disponible mais non fonctionnel | Disponible et fonctionnel | |
| --- | --- | --- | --- | --- |
|  |  |  | Oui | Nombre |
| Table d’examen |  |  |  |  |
| Table d’accouchement |  |  |  |  |
| Stéthoscope obstétrical |  |  |  |  |
| Tensiomètre |  |  |  |  |
| Thermomètre |  |  |  |  |
| Mètre ruban |  |  |  |  |
| Spéculum |  |  |  |  |
| Boîte d’accouchement |  |  |  |  |

12. Est-ce qu’il y a souvent plus d’une femme en travail à la fois dans le CSPS ?  OUI  NON

**Salle de suite de couches**

| 13. Éléments  (observer) | Nombre total | Nombre selon l’état * | | |
| --- | --- | --- | --- | --- |
|  |  | Bon | Moyen | Mauvais |
| Lits avec matelas |  |  |  |  |
| Toilettes à proximité de salle de suite de couches |  |  |  |  |
| Sources d’eau potable dans l’enceinte du CSPS |  |  |  |  |

* État des lits :

- Matelas recouvert de skaï= bon

- Skaï troué par endroits= moyen

- Pas de skaï = mauvais

État des toilettes : jugé selon la propreté

Source d’eau :

- Si non fonctionnelle, noter zéro

- Robinet ou pompe = bon

- Puits grand diamètre aménagé = moyen

- Puits grand diamètre non aménagé =mauvais

14. Le jour de la visite, le nombre de lits est-il suffisant pour le nombre de femmes en suites de couches ?  OUI  NON

**Moyens de référence-évacuation**

15. Le CSPS dispose-t-il d’un moyen de transport pour les évacuations de parturientes :

| Ambulance |  |  |  |
| --- | --- | --- | --- |
| Moto ambulance |  |  |  |
| Pas de moyen de transport |  | (si aucun moyen de transport : aller directement à la question 21) | |

16. Durant les 3 derniers mois, est-il arrivé que tous ces véhicules soient en panne ?

OUI (nombre de jours : ………)  NON

17. Ces véhicules sont-ils utilisés pour le transport de femmes en travail depuis les villages vers le CSPS ?  OUI  NON

18. Vers quel(s) CMA se font les évacuations ?

| CMA du district |  |
| --- | --- |
| Autre CMA (préciser : ……………………..) |  |

19. Quelle est la durée moyenne pour faire le trajet jusqu’au CMA ?

|  | Saison sèche | Hivernage |
| --- | --- | --- |
| CMA du district |  |  |
| Autre CMA (préciser: ………………..) |  |  |

20. La femme ou sa famille doivent-elles effectuer un paiement avant que l’évacuation soit faite?

|  | Oui | Non | Sans objet  (pas d’évacuations sur ces trajets) |
| --- | --- | --- | --- |
| Évacuation depuis villages vers CSPS |  |  |  |
| Évacuation depuis CSPS vers CMA du district |  |  |  |
| Évacuation depuis CSPS vers autre CMA |  |  |  |

21. Moyens de communication pour joindre le CMA du district en cas d’urgence :

| Élément | Non disponible ou non fonctionnel | Disponible par intermittence* | Disponible en permanence |
| --- | --- | --- | --- |
|
| RAC du CSPS |  |  |  |
| RAC du CMA |  |  |  |
| Téléphone fixe |  |  |  |
| Téléphone mobile appartenant au CSPS |  |  |  |
| Téléphones mobiles appartenant aux membres du personnel |  |  |  |

* = périodes sans disponibilité durant les 3 mois passés, ou disponibilité seulement certains jours ou heures.

22. Quelle est la durée moyenne pour mobiliser l’ambulance du CMA (délai entre la réalisation de l’appel et l’arrivée de l’ambulance au CSPS) :

Saison sèche : …………………..

Hivernage : ……………………

(Si durée moyenne variable : donner une « fourchette » minimum et maximum pour chaque saison).

**Tarification**

23. Pratiquez-vous un système de paiement forfaitaire pour l’accouchement :

|  | Forfait (oui / non) | Si oui : montant en FCFA |
| --- | --- | --- |
| Accouchement eutocique |  |  |
| Accouchement dystocique |  |  |

24. Si paiement non forfaitaire, quels sont les tarifs :

|  | Accouchement simple | Accouchement avec épisiotomie |
| --- | --- | --- |
| Acte |  |  |
| Kit (ou montant moyen payé pour les consommables) |  |  |
| Mise en observation |  |  |
| Montant moyen des ordonnances après accouchement |  |  |

25. Moment du paiement par la femme ou sa famille :

- Si tarification par forfait :  Paiement avant accouchement  Paiement après accouchement

- Si tarification hors forfait, cocher les réponses appropriées :

|  | Avant accouchement | Après accouchement |
| --- | --- | --- |
| Paiement de l’acte |  |  |
| Paiement du kit d’accouchement |  |  |

**Activité** **du CSPS**

**À relever dans les rapports mensuels du CSPS**

26. Nombre d’accouchements durant les 3 derniers mois

(Relever seulement le nombre d’accouchements en maternité)

| Mois | Nombre |
| --- | --- |
| Mois 1 |  |
| Mois 2 |  |
| Mois 3 |  |
| **Total** |  |

27. Nombre d’évacuations de parturientes durant les 3 derniers mois

| Mois | Nombre |
| --- | --- |
| Mois 1 |  |
| Mois 2 |  |
| Mois 3 |  |
| **Total** |  |
